# Rapid treatment center for depression in China: constructive reflections and transnational implications

**DOI:** 10.3389/fpsyt.2025.1582312

**Published:** 2025-06-16

**Authors:** Zhan-Ming Shi, Xing-Bing Huang, Yan-Ling Zhou, Yu-Ping Ning, Gabor S. Ungvari, Yu-Tao Xiang, Wei Zheng

**Affiliations:** 1 Department of Psychiatry, Chongqing Jiangbei Second Hospital, Chongqing Jiangbei Mental Health Center, Chongqing, China; 2 Department of Psychiatry, The Affiliated Brain Hospital, Guangzhou Medical University, Guangzhou, China; 3 Key Laboratory of Neurogenetics and Channelopathies of Guangdong Province and the Ministry of Education of China, Guangzhou Medical University, Guangzhou, China; 4 Section of Psychiatry, University of Notre Dame Australia, Fremantle, WA, Australia; 5 Division of Psychiatry, School of Medicine, University of Western Australia, Perth, WA, Australia; 6 Unit of Psychiatry, Department of Public Health and Medicinal Administration, & Institute of Translational Medicine, Faculty of Health Sciences, University of Macau, Macao, Macao SAR, China; 7 Centre for Cognitive and Brain Sciences, University of Macau, Macao, Macao SAR, China

**Keywords:** major depressive disorder, electroconvulsive therapy, ketamine, esketamine, esketamine nasal spray, magnetic seizure therapy, Stanford Neuromodulation Therapy, rapid treatment center

## Abstract

Enhancing the early diagnosis and standardized treatment of major depressive disorder (MDD), as advocated by the National Health Commission of China, is a key priority in the mental health initiatives of the nation. To achieve this, numerous evidence-based rapid treatment centers for MDD have been established or are currently under development across the country. These centers focus on the following primary treatment modalities for depression: electroconvulsive therapy (ECT), intravenous ketamine/esketamine, esketamine nasal spray, magnetic seizure therapy (MST), and Stanford Neuromodulation Therapy (SNT). This policy and practice review explored the application and modification of these techniques in treating depression in China, addressing their strengths and shortcomings. With particular focus on China’s rapid antidepressant treatment strategies (e.g., ECT, intravenous ketamine/esketamine, esketamine nasal spray, MST, SNT), the current policy and practice review described their potential to speed up recovery thereby providing valuable insights for other countries and regions.

## 1 Introduction

Major depressive disorder (MDD) is one of the most prevalent mental illnesses, characterized by persistent low mood and diminished interest among several other symptoms (1). In severe cases, patients may exhibit serious psychological and behavioral manifestations, such as hallucinations, delusions, self-harm, and suicidal tendencies (2). The prevalence and incidence of MDD have increased recently, particularly in adolescents, probably due to the influence of the COVID-19 pandemic (3, 4).

To address this escalating trend in MDD prevalence, the National Health Commission of China has devised a blueprint of a specialized service aimed at preventing and treating MDD. For instance, the ‘Healthy China 2030’ initiative called for enhancing the early detection and timely intervention for MDD. The ‘Specialized Service Program for Depression Prevention and Treatment’ emphasized the necessity of introducing early diagnostic protocols, standardizing treatment modalities, and intensifying intervention strategies particularly for pivotal cohorts such as children, adolescents and expectant and postnatal mothers. To date, numerous general hospitals and mental health facilities across China have established expedited treatment centers for MDD, predominantly focusing on these cutting-edge treatment methods: electroconvulsive therapy (ECT), intravenous ketamine/esketamine, esketamine nasal spray, magnetic seizure therapy (MST), and Stanford Neuromodulation Therapy (SNT). The prompt alleviation of symptoms rapidly reduces patients’ suffering, bolsters compliance and enhances overall quality of life (5).

Although the current mainstream rapid antidepressant methods encompass ECT, intravenous ketamine/esketamine, esketamine nasal spray, MST and SNT, ECT remains the predominant and favored approach for swift antidepressant and anti-suicidal interventions in China. In alignment with the objective of rapid antidepressive treatment, each of these major techniques demonstrates distinct advantages: ECT and MST work through immediate neuromodulation, intravenous ketamine/esketamine and esketamine nasal spray act via N-methyl-D-Aspartate (NMDA) receptor antagonism, and SNT operates by regulating biological rhythm. Taken together, these techniques constitute the multimodal intervention framework employed in China’s rapid depression treatment centers. ECT is particularly applicable to patients with MDD in special populations, including adolescents, elderly individuals, and patients in perinatal period (6). Currently, research and clinical application of intravenous ketamine/esketamine, esketamine nasal spray, MST, and SNT are predominantly administered to adult MDD patients, with insufficient evidence to support their widespread use in the above-mentioned special populations.

This policy and practice review examined the localization and clinical implementation of these rapid antidepressant techniques in China, with dual emphasis on exploring service delivery shortcomings and prioritizing future research needs. Sharing the experiences of China in rapid antidepressive treatment using these techniques (e.g., ECT, intravenous ketamine/esketamine, esketamine nasal spray, MST, SNT) could offer valuable reference models and strategies for other nations and regions.

## 2 Methods

As a policy and practice review focusing on experiences in implementation rather than synthesis of evidence, this work did not employ any formal search strategy or inclusion/exclusion criteria for eligibility.

## 3 Actionable recommendations

### 3.1 Electroconvulsive therapy

ECT has a history of over 80 years as one of the most efficient interventions for MDD. Its use is more prevalent in China than in most other countries (7), with China probably having the largest cohort of patients receiving ECT globally (8). ECT is used as a pivotal clinical recourse for patients with schizophrenia, bipolar disorder and MDD, especially those resistant to conventional therapies and displaying high suicide risks (9). Because of its relatively moderate efficacy rate of 58% and 70%, for those with and without medication failure, respectively (10) and known neurocognitive side effects (11), there are ongoing efforts aiming at refining the clinical application of ECT from various perspectives, including anesthetic selection, stimulation dosage, electrode placement and apnoeic oxygenation.

Concerning anesthetic practices, the combined use of intravenous ketamine/esketamine or their standalone administration emerged as prevalent strategies to enhance ECT’s effectiveness. A recent randomized controlled trial (RCT) showed that intravenous esketamine as an anesthetic for ECT yielded superior antidepressant outcome with comparable neurocognitive effects compared to ECT with propofol anesthesia, with higher MATRICS Consensus Cognitive Battery scores in processing speed, working memory, verbal learning, and visual learning (12). Moreover, a meta-analysis suggested that combining intravenous ketamine with other anesthetics may have transient advantages in ameliorating depressive symptoms during the initial phase of ECT (13).

Recent clinical observations have highlighted the significant and immediate antidepressant impact of low-dose ECT (near the seizure threshold) in MDD (14), a finding confirmed by a RCT in China (15). Even in cases of treatment-resistant depression (TRD), nonconvulsive electrotherapy (NET) has demonstrated a comparable therapeutic outcome, yielding response rates as high as 60.0% (16). A systematic review indicated that NET’s antidepressant efficacy for MDD is similar to ECT, albeit with notably lesser neurocognitive impairments (17). However, the current Chinese Expert Consensus on ECT does not mention NET for routine clinical application (6). As for the knowledge of, and attitude towards ECT, a recent study found that only 55.4% and 37.0% of caregivers and patients received any pre-treatment education, respectively (18); caregivers were provided with significantly more comprehensive ECT information than patients. Significantly more caregivers than patients regarded ECT as effective (71.7% versus 53.3%; *p* < 0.05) and approximately half of both groups considered it safe (18).

Furthermore, a novel approach termed hybrid-ECT has been introduced by Rong et al., wherein MDD patients undergo an initial trio of ECT sessions followed by NET instead of continuing standard sessions of ECT (19). Hybrid-ECT may retain ECT’s early treatment benefits while capitalizing on NET’s advantage of minimizing side effects in the longer term. A RCT assessing hybrid-ECT for MDD showed equivalent antidepressant effects to standard ECT but with fewer adverse events including neurocognitive impairment (20).

Innovations in electrode placements for ECT, ranging from traditional setups like right unilateral, bitemporal, and bifrontal to newer configurations, such as focal, electrically-administered seizure therapy (21), left anterior-right temporal (22), left unilateral (23) and others, have emerged. However, comprehensive research on these novel electrode placements remains relatively scarce, with some (e.g., fronto-frontal, fronto-parietal, temporo-parietal) currently in the computational modeling phase (24).

Transnasal Humidified Rapid-Insufflation Ventilatory Exchange (THRIVE) is a secure, effective new technique now integrated into ECT procedures (25–27). THRIVE facilitates oxygen delivery with features like operational simplicity, temperature stability and humidity control, thereby reducing the risk of gastric reflux and extending safe apnea durations. Patients have reported positive experiences, propelling THRIVE into widespread adoption in general anesthesia practices in recent years (28).

### 3.2 Intravenous ketamine/esketamine and esketamine nasal spray

Ketamine, a racemic mixture comprising both S- and R-enantiomers, and esketamine, the purified S-enantiomer, share a core mechanism as NMDA receptor antagonists, inducing rapid synaptic plasticity via glutamate surge, alpha-Amino-3-hydroxy-5-methyl-4-isoxazolepropionic acid receptor activation, and downstream brain-derived neurotrophic factor (BDNF)/mammalian target of rapamycin signaling (29). The key difference lies in esketamine’s higher affinity for NMDA receptors due to its S-isomer configuration, potentially enhancing its antidepressant potency at lower doses. While both drugs produce rapid-onset effects (within hours), ketamine’s metabolites (e.g., hydroxynorketamine) may contribute to its antidepressant action through non-NMDA pathways, whereas esketamine’s effects are more directly tied to NMDA blockade (30, 31). Intravenous ketamine has been approved for use as a sedative, analgesic and general anesthetic since 1970 by the Food and Drug Administration (FDA) (32). Intravenous administration of ketamine/esketamine at sub-anesthetic doses has demonstrated rapid antidepressant and anti-suicidal effects in patients with MDD (33, 34). Both single and repeated doses of ketamine have shown robust antidepressant effect in patients with MDD (35). While the rapid treatment centers for depression in China have adopted this technology and achieved promising results (36–39), intravenous ketamine/esketamine still lack approval for antidepressant indications by the China Food and Drug Administration.

Various methods of administering ketamine/esketamine such as intravenous, intranasal, oral, intramuscular, and subcutaneous have been tried (40). However, there have been no RCTs directly comparing the antidepressant efficacy of different routes of ketamine/esketamine administration. Intravenous and intranasal administration are the most common therapeutic routes. Kheirabadi et al.’s study suggested that oral and intramuscular ketamine may have equivalent antidepressant and greater anti-suicidal effects compared to ECT but with fewer adverse neurocognitive effects (41). A meta-analysis of three RCTs found a significant antidepressant effect of oral ketamine compared to placebo (42). Repeated subcutaneous ketamine injections were found well tolerated and more effective than midazolam, with acute adverse dissociative and other psychopathological symptoms resolved within 2 hours (43). Midazolam is rarely used for anaesthesia for ECT in most Western countries, but not in China (44). Importantly, plasma levels from subcutaneous ketamine administration are comparable to those of intravenous and intramuscular ketamine, but with minimal side effects, suggesting that subcutaneous ketamine is a practical and effective treatment approach (45). In 2019, the FDA approved esketamine nasal spray in combination with oral antidepressants for treating adults with TRD (46) and in 2020, for treating adults with MDD and acute suicidal ideation or behavior (47). In 2023, the China Food and Drug Administration approved it for the same uses in China as in the USA, although its application remains limited. According to the clinical application guidance for esketamine in China (48), intravenous esketamine (0.2-0.4 mg per kilogram of body weight over 40 minutes) can be used for the rapid treatment of MDD. It has been suggested that combining two drugs with different neurochemical pathways can have a synergistic effect, resulting in a faster and stronger antidepressant action (49). By combining ECT with intravenous ketamine/esketamine, a few psychiatric hospitals established an ultra-rapid antidepressant center in 2024. However, the promotion and utilization of intravenous ketamine/esketamine and esketamine nasal spray for MDD in China are still suboptimal (49).

### 3.3 Magnetic seizure therapy

MST delivers high-intensity magnetic stimulation to induce therapeutic epileptic seizures in specific areas of the brain’s vertex in patients with MDD aiming to ameliorate depressive symptoms. Compared to ECT, MST-induced seizures are more localized, potentially resulting in fewer neurocognitive side effects and quicker recovery (50) and as such, MST is viewed as a plausible alternative to ECT (51). Capitalizing on its advantageous profile of comparable antidepressant efficacy to ECT (52), a team at Beijing Anding Hospital of Capital Medical University have adopted an accelerated MST protocol termed daily MST, demonstrating rapid antidepressant effects (53). MST has been emerging as a novel treatment method in clinical practice, showcasing significant antidepressant efficacy in China (54, 55).

The pathomechanism underlying MST is still not well elucidated. Neuroimaging studies utilizing positron emission tomography/computer tomography scans have revealed post-treatment increases in metabolism in the basal ganglia, orbitofrontal cortex, medial frontal cortex, bilateral frontal cortex and dorsolateral prefrontal cortex, alongside a decrease in the left striatum (56, 57). Discrepancies in brain activation within the ventral anterior cingulate have been noted between MDD responders and non-responders (56). Resting-state functional magnetic resonance imaging (fMRI) and structural MRI studies have indicated enhanced functional connectivity between the subgenual anterior cingulate cortex (sgACC) and the parietal cortex following MST treatment, correlating positively with clinical improvement. Changes in grey matter volume have been observed in the bilateral parietal cortex but not associated with treatment outcomes (58). Moreover, MST has been shown to selectively modulate brain network dynamics for therapeutic benefits (59–61) and to enhance aperiodic neural activity linked to increased neural inhibition (62). In terms of neuroplasticity, MST can boost neuroplasticity in excitatory and inhibitory circuits originating from the left dorsolateral prefrontal cortex (DLPFC), and it also diminishes long-interval cortical inhibition over the right frontal cortex and induces neuroplastic changes in the frontal cortex, possibly through long-term potentiation - like mechanisms (63). Furthermore, MST can facilitate the central motor pathway elevating motor cortex excitability (54). These findings suggest a potentially shared neural mechanism of action between ECT and MST, underscoring MST’s promise as an effective MDD treatment, particularly for non-responsive or patients intolerant to conventional antidepressants. Nonetheless, the mechanism of action of MST warrants further investigation, with additional clinical trials to validate its efficacy and safety. Given its minimal impact on neurocognitive function, MST holds promise as a rapid antidepressant modality.

### 3.4 Stanford Neuromodulation Therapy

Traditional transcranial magnetic stimulation (TMS) is imprecise to stimulate localized targets (64), it demonstrates modest efficacy (65), and requires long intervention periods (e.g., 37.5 minutes per session, 5 sessions weekly spanning 4–6 weeks) (66). In recent years, numerous optimization strategies for TMS have emerged, including theta burst stimulation (TBS) (67) and deep TMS (68). Accelerated intermittent TBS (aiTBS), a variant of TBS involving multiple sessions distributed across several days has gained widespread clinical utilization, reducing the treatment duration and total impulse count and demonstrating swift antidepressant and anti-suicidal effects in MDD (69, 70).

Another TBS form, SNT, has also exhibited rapid antidepressant effects (71). Notably, two open-label studies and one RCT have indicated a remission rate of approximately 90.0%, even in treatment-resistant patients, without significant adverse events (72–74). A few hospitals in China offer original Stanford protocol for MDD (75–77). The standard SNT protocol includes: 1) the adoption of an efficient repetitive TMS form, known as iTBS; 2) implementation of multiple iTBS sessions daily at optimally spaced intervals; 3) the application of a higher cumulative pulse dose of stimulation; and 4) personalized targeting for stimulating the left DLPFC to the sgACC circuit (72). However, in the absence of functional connectivity-guided targeting in most hospital settings, clinicians typically resort to standard aiTBS approaches (69, 78). Moreover, the evidence supporting SNT predominantly derives from Caucasian populations, leaving the efficacy and safety of these parameters in Asians still uncertain. Further investigations are warranted to ascertain the occurrence of treatment-emergent (hypo)mania in MDD and bipolar depression patients at intensified and concentrated treatment doses administered with SNT.

## 4 Discussion

### 4.1 Clinical/procedural issue

China’s experience in implementing rapid treatment for depression may provide valuable insights for other nations seeking to establish such services. Specifically, clinicians in China have developed innovative modifications to ECT, pioneered accelerated MST protocols. A unique aspect involves close collaboration between psychiatrists and anesthesiologists to facilitate efficient treatment delivery. For resource-limited settings where SNT is impractical, aiTBS may serve as a viable alternative, demonstrating comparable antidepressant efficacy. These adaptations highlight how context-specific innovations could enhance accessibility to rapid antidepressant interventions across diverse healthcare conditions. The optimal therapeutic dosage of intravenous ketamine/esketamine and esketamine nasal spray may vary across different countries and regions. For instance, the commonly used dose of intravenous ketamine in China is 0.5 mg per kilogram of body weight administered over 40 minutes (79), with adjustments required based on individual patient factors and local clinical guidelines.

To date, China has established specialized management and treatment programs for patients with severe mental disorders, incorporating regular follow-up visits, treatment assistance, and rehabilitation guidance, with particular emphasis on integrated whole-course disease management services (80). However, such comprehensive programs have not yet been systematically implemented for patients with MDD. This service gap could lead to suboptimal treatment adherence following acute-phase therapy, insufficient rehabilitative support, and ultimately hinders patients’ successful social reintegration.

Several factors limit the widespread use of intravenous ketamine/esketamine and esketamine nasal spray in China. First, as a psychotropic anesthetic, intravenous ketamine/esketamine and esketamine nasal spray is strictly regulated in China and must be administered under medical supervision in designated medical facilities. This strict regulation restricts its use. Furthermore, China currently lacks a formal expert consensus or guidelines on intravenous ketamine/esketamine and esketamine nasal spray therapy for patients with MDD, thereby failing to establish standardized protocols for off-label use, signing of informed consent, scheduling of intravenous ketamine/esketamine and esketamine nasal spray, risk assessment, systematic adverse effect evaluation using validated rating scales (81), and longitudinal monitoring requirements. Considering the side effects of intravenous ketamine/esketamine and esketamine nasal spray and the distinctive characteristics of adolescent brain development and its susceptibility to drug reactions, the utilization of intravenous ketamine/esketamine and esketamine nasal spray in adolescents needs more rigorous evaluation and monitoring. Second, it is still unknown whether intravenous ketamine/esketamine and esketamine nasal spray have addictive properties as an adverse effect of the treatment. While the use of intravenous ketamine/esketamine and esketamine nasal spray in China is restricted to medical institutions under direct supervision of healthcare professionals, a policy designed to minimize abuse potential, significant gaps remain in treatment monitoring. The lack of a centralized tracking system allows patients with MDD to seek treatment from different hospitals during a short period without restrictions, which raises serious concerns about potential abuse. Patients with addictive tendencies in China must undergo treatment in drug rehabilitation centers. Fear of addiction and adverse dissociative and other psychopathological symptoms have led some MDD patients to prefer other antidepressant interventions. The long-term safety of intravenous ketamine/esketamine and esketamine nasal spray requires further verification. Finally, the cost of esketamine nasal spray is significantly higher than most other antidepressants and limited medical insurance coverage limits its widespread use in China.

At present, MST lacks an independent fee code within the Chinese medical insurance system. Several hospitals either adhere to the billing standards of TMS or ECT or offer free treatment to MDD patients, thereby hindering the dissemination and utilization of MST. Without formal insurance coverage, most hospitals lack incentives to invest in MST equipment, and patients usually face prohibitive out-of-pocket costs, limiting accessibility to MST despite its therapeutic potential. Moreover, the considerable size and high cost of MST equipment impedes its widespread adoption and application. Several key challenges must be addressed before MST can be established as a viable ECT alternative. First, further research is needed to optimize stimulus parameters, including location and frequency, for treating major mental disorders. Second, while the restricted stimulation range of MST aids in investigating treatment mechanisms, future studies should comprehensively explore these mechanisms, encompassing neurotransmitter activity of, for instance,5-hydroxytryptamine, norepinephrine, and dopamine, neuroplasticity (e.g., BDNF), neuroendocrine functions (e.g., hypothalamic-pituitary-adrenal axis, hypothalamic-pituitary-thyroid axis, and hypothalamic-pituitary-gonadal axis), and neuroimaging (e.g., diffusion tensor imaging, voxel-based morphometry, and magnetic resonance spectroscopy). This approach will yield valuable insights for shaping future treatment strategies.

### 4.2 Comparisons of rapid antidepressant treatments

A recent meta-analysis supported the use of ECT over ketamine for inpatients (82). Conversely, another meta-analysis of RCTs comparing the efficacy of ECT with ketamine revealed no significant disparity between the two treatments (83). Despite the rapid antidepressant effects of these methods, there is a relative dearth of head-to-head RCTs directly comparing them, for instance, ECT versus SNT. It is essential to take into account that ketamine and esketamine can be delivered by various routes, with differences in drug absorption, distribution, metabolism and excretion among them. Also, it is crucial to discern the variances in rapid antidepressant efficacy between ECT and ketamine/esketamine based on different administration routes (84, 85).

While ECT is sometimes combined with intravenous ketamine/esketamine for prompt antidepressant care, the optimal order, interval and frequency of ECT and intravenous ketamine/esketamine administration remain uncertain necessitating further exploration. Establishing a nationwide monitoring network to document the frequency of intravenous ketamine/esketamine and esketamine nasal spray administration in MDD patients and monitor potential addiction risks is imperative.

Regarding ECT versus MST, a meta-analysis of four RCTs (52) and a recent RCT (86) consistently demonstrated that MST’s efficacy was comparable to that of ECT. ECT, the globally recommended gold standard for rapid antidepressant treatment (9), yields a lower efficacy rate than SNT (approximately 60.0% versus 90.0%), underscoring the urgent need for exploring the efficacy and safety of ECT in comparison to SNT.

Furthermore, investigating the optimal treatment parameters (e.g., frequency and dosage) of these rapid antidepressant methods for MDD and utilizing artificial intelligence (AI) to forecast changes in depressive symptoms would enable personalized decision-making in clinical practice. To implement AI-based symptom prediction in clinical settings, data requirements include structured clinical variables (e.g., sociodemographics, psychiatric history, comorbidities, prior treatments), standardized psychometric assessments, treatment response metrics (e.g., symptom reduction, remission), longitudinal follow-up data, and, ideally, biomarkers (e.g., neuroimaging and genetic markers), with assessments conducted by psychiatrists and clinical psychologists to ensure reliability (87).

### 4.3 General/systemic deficiencies

While a variety of rapid antidepressant therapies have been implemented in most cities of China, significant regional disparities persist in mental healthcare infrastructure. For instance, 12.3% of county-level districts lack mental health institutions at all and 31.1% have no psychiatric beds; these deficiencies are concentrated predominantly in the central and western regions of China. Western China demonstrates particularly obvious resource gaps, having approximately 4-fold fewer open psychiatric beds, psychiatrists, and psychiatric nurses compared to central regions, and 7-11 fold fewer than eastern regions (88). Furthermore, less than 30.0% of mental health institutions nationwide have established psychiatric rehabilitation services, with 66.4% providing rehabilitation services exclusively for inpatients (88). This severely inadequate rehabilitation infrastructure fails to meet service demands, particularly for community-dwelling patients requiring continued care.

There are also significant disparities in access to MDD treatment between rural and urban areas in China. In rural regions, only 5.1% of patients with depressive disorders received any form of healthcare treatment, compared to 8.9% in urban areas (89). This gap exists mainly due to fewer mental health facilities and lower incomes for rural patients. Additionally, mental health services are mainly located in urban psychiatric hospitals, making travel and relative treatment costs prohibitive for rural patients.

### 4.4 Ethical issues

Informed consent to ECT for depression mandates full disclosure to the patients’ primary guardian, including details on treatment principles, indications, efficacy, and risks. In cases that severe symptoms necessitate urgent ECT but guardians are unavailable, hospitals need to seek legal alternative consent following local regulations. Due to cultural norms emphasizing familial involvement and current and past sociocultural dynamics, most depression patients in China usually rely on family caregivers. However, the overriding decision-making authority often retained by relatives compromises patient autonomy. This situation is not unique to China, patients’ autonomy is also compromised in Western societies. In Western contexts, the guardian (if appointed) and the court typically give permission to proceed with ECT, rather than the family, although the family’s viewpoint is taken into account. This highlights that China’s informed consent model, influenced by its specific legal and cultural context, may not be directly applicable in other settings.

### 4.5 Limitations

This policy and practice review has several limitations. First, it did not fully frame the findings as a comprehensive report on the development, implementation, and evaluation of rapid treatment centers for depression in China. Second, while implementation science frameworks have been widely applied in health services research, their use in psychiatry, especially with respect to these rapid antidepressant treatment strategies, remains notably limited in China. Future research should apply implementation science frameworks to bridge the gap between evidence-based interventions and routine clinical practice, thereby establishing an all-inclusive implementation model.

## 5 Conclusion

This policy and practice review provides a comprehensive examination of rapid treatment centers for depression in China focusing on the constructive reflections and transnational implications of the following rapid antidepressant therapies: ECT, intravenous ketamine/esketamine, esketamine nasal spray, MST, and SNT. Based on the findings of this policy and practice review, we propose the following: (1) for depressed patients with suicidal risk, ECT and esketamine nasal spray are preferentially recommended as first-line rapid antidepressant treatment; (2) to create a national registry to monitor intravenous ketamine/esketamine and esketamine nasal spray utilization while ensuring safety compliance. Although numerous evidence-based rapid treatment centers for MDD have been established or are currently under development across the country, the following concerns should be addressed in the future: (1) to assess the long-term safety, addictive properties, and neurobiological consequences of intravenous ketamine/esketamine and esketamine nasal spray in treating MDD; (2) to evaluate whether MST could serve as a viable alternative to ECT for rapid antidepressant effects in MDD; (3) to evaluate the efficacy, safety, and acceptability of standardized SNT protocols across China’s diverse clinical settings to establish locally optimized treatment parameters; (4) to determine which rapid antidepressant interventions have the most effective antidepressive and anti-suicide outcomes; (5) to enhance the clinical applicability of these rapid antidepressant interventions in bipolar depression or special populations (e.g., adolescents and perinatal women).

## References

[1] Beğinoğlu ÖChecktae Asoğlu M . Evaluation of thiol/disulfide interrelation in major depressive disorder. Alpha Psychiatry. (2023) 24:283–7. doi: 10.5152/alphapsychiatry.2023.231277 PMC10837513 38313439

[2] Liu J Guan J . Consanguinity and nonsuicidal self-injury in depressed patients: new risk factors and risk prediction models. Alpha Psychiatry. (2024) 25:82–7. doi: 10.5152/alphapsychiatry.2024.231223 PMC11114175 38799484

[3] COVID-19 Mental Disorders Collaborators . Global prevalence and burden of depressive and anxiety disorders in 204 countries and territories in 2020 due to the COVID-19 pandemic. Lancet. (2021) 398:1700–12. doi: 10.1016/s0140-6736(21)02143-7 PMC8500697 34634250

[4] Racine N McArthur BA Cooke JE Eirich R Zhu J Madigan S . Global prevalence of depressive and anxiety symptoms in children and adolescents during COVID-19: a meta-analysis. JAMA Pediatr. (2021) 175:1142–50. doi: 10.1001/jamapediatrics.2021.2482 PMC8353576 34369987

[5] Aaronson ST van der Vaart A Miller T LaPratt J Swartz K Shoultz A . Single-dose synthetic psilocybin with psychotherapy for treatment-resistant bipolar type II major depressive episodes: a nonrandomized open-label trial. JAMA Psychiatry. (2024) 81:555–62. doi: 10.1001/jamapsychiatry.2023.4685 PMC10701666 38055270

[6] Chinese Association of Neurological Regulation Committee for Electroconvulsive Therapy and Nerve Stimulation Chinese Association of Sleep Committee for Mental Psychology, Chinese Association of Anesthesiology . Expert consensus on modified electroconvulsive therapy (2019) (In Chinese). Trans Med J. (2019) 8:129–34. doi: 10.3969/j.issn.2095-3097.2019.03.001

[7] Wang ZM Zhu H Pan YL Chiu HF Correll CU Ungvari GS . Electroconvulsive therapy and its association with demographic and clinical characteristics in Chinese psychiatric patients. J ECT. (2015) 31:114–8. doi: 10.1097/yct.0000000000000181 25203288

[8] Tang YL Jiang W Ren YP Ma X Cotes RO McDonald WM . Electroconvulsive therapy in China: clinical practice and research on efficacy. J ECT. (2012) 28:206–12. doi: 10.1097/YCT.0b013e31825957b1 22801297

[9] Thirthalli J Sinha P Sreeraj VS . Clinical practice guidelines for the use of electroconvulsive therapy. Indian J Psychiatry. (2023) 65:258–69. doi: 10.4103/Indianjpsychiatry.Indianjpsychiatry_491_22 PMC10096214 37063631

[10] Haq AU Sitzmann AF Goldman ML Maixner DF Mickey BJ . Response of depression to electroconvulsive therapy: a meta-analysis of clinical predictors. J Clin Psychiatry. (2015) 76:1374–84. doi: 10.4088/JCP.14r09528 26528644

[11] Nuninga JO Claessens TFI Somers M Mandl R Nieuwdorp W Boks MP . Immediate and long-term effects of bilateral electroconvulsive therapy on cognitive functioning in patients with a depressive disorder. J Affect Disord. (2018) 238:659–65. doi: 10.1016/j.jad.2018.06.040 29966930

[12] Zeng QB Zou DC Huang XB Shang DW Huang X Yang XH . Efficacy and safety of esketamine versus propofol in electroconvulsive therapy for treatment-resistant depression: A randomized, double-blind, controlled, non-inferiority trial. J Affect Disord. (2024) 368:320–8. doi: 10.1016/j.jad.2024.09.038 39265871

[13] Zheng W Li XH Zhu XM Cai DB Yang XH Ungvari GS . Adjunctive ketamine and electroconvulsive therapy for major depressive disorder: A meta-analysis of randomized controlled trials. J Affect Disord. (2019) 250:123–31. doi: 10.1016/j.jad.2019.02.044 30852364

[14] Lapidus KA Shin JS Pasculli RM Briggs MC Popeo DM Kellner CH . Low-dose right unilateral electroconvulsive therapy (ECT): effectiveness of the first treatment. J ECT. (2013) 29:83–5. doi: 10.1097/YCT.0b013e31827e0b51 23449042

[15] Li W Ji C Yang K Cai H Wang X Wei X . Evaluation of efcacy and safety about sub-threshold modifed electroconvulsive therapy for depression (In Chinese). Chin J Psychiatry. (2020) 53:42–8. doi: 10.3760/cma.j.issn.1006-7884.2020.01.008

[16] Zheng W Jiang ML He HB Li RP Li QL Zhang CP . A preliminary study of adjunctive nonconvulsive electrotherapy for treatment-refractory repression. Psychiatr Q. (2021) 92:311–20. doi: 10.1007/s11126-020-09798-3 32661940

[17] Cai DB Zhou HR Liang WN Gu LM He M Huang X . Adjunctive nonconvulsive electrotherapy for patients with depression: a systematic review. Psychiatr Q. (2021) 92:1645–56. doi: 10.1007/s11126-021-09936-5 34159503

[18] Deng CJ Nie S Mai JX Huang X Huang XB Zheng W . Electroconvulsive therapy knowledge and attitudes among patients and caregivers in South China: a preliminary study. Front Psychiatry. (2023) 14:1145301. doi: 10.3389/fpsyt.2023.1145301 36993925 PMC10040676

[19] Rong H Xu SX Zeng J Yang YJ Zhao J Lai WT . Study protocol for a parallel-group, double-blinded, randomized, controlled, noninferiority trial: the effect and safety of hybrid electroconvulsive therapy (Hybrid-ECT) compared with routine electroconvulsive therapy in patients with depression. BMC Psychiatry. (2019) 19:344. doi: 10.1186/s12888-019-2320-3 31694611 PMC6836661

[20] Zhang JY Xu SX Zeng L Chen LC Li J Jiang ZY . Improved safety of hybrid electroconvulsive therapy compared with standard electroconvulsive therapy in patients with major depressive disorder: a randomized, double-blind, parallel-group pilot trial. Front Psychiatry. (2022) 13:896018. doi: 10.3389/fpsyt.2022.896018 35677877 PMC9168000

[21] Sahlem GL McCall WV Short EB Rosenquist PB Fox JB Youssef NA . A two-site, open-label, non-randomized trial comparing focal electrically-administered seizure therapy (FEAST) and right unilateral ultrabrief pulse electroconvulsive therapy (RUL-UBP ECT). Brain Stimul. (2020) 13:1416–25. doi: 10.1016/j.brs.2020.07.015 PMC7500956 32735987

[22] Weiss AM Hansen SM Safranko I Hughes P . Effectiveness of left anterior right temporal electrode placement in electroconvulsive therapy: 3 case reports. J ECT. (2015) 31:e1–3. doi: 10.1097/yct.0000000000000136 24831996

[23] Kellner CH Farber KG Chen XR Mehrotra A Zipursky GDN . A systematic review of left unilateral electroconvulsive therapy. Acta Psychiatr Scand. (2017) 136:166–76. doi: 10.1111/acps.12740 28422271

[24] Martin D Lin F Bai S Moffa A Taylor R . A systematic review and computational modelling analysis of unilateral montages in electroconvulsive therapy. Acta Psychiatr Scand. (2019) 140:408–25. doi: 10.1111/acps.13089 31419305

[25] Jonker Y Rutten DJ van Exel ER Stek ML de Bruin PE Huitink JM . Transnasal humidified rapid-insufflation ventilatory exchange during electroconvulsive therapy: a feasibility study. J ECT. (2019) 35:110–4. doi: 10.1097/yct.0000000000000556 30461537

[26] Vaithialingam B Bansal S Ramesh VJ Muthuchellappan R . Trans-nasal humidified rapid insufflation ventilatory exchange (THRIVE) ventilation during electroconvulsive therapy (ECT) for a pregnant patient- a novel technique. Asian J Psychiatr. (2022) 70:103023. doi: 10.1016/j.ajp.2022.103023 35183042

[27] Vaithialingam B Bansal S Muthuchellappan R Thirthalli J Chakrabarti D Venkatapura RJ . Comparison of hands-free trans-nasal humidified rapid insufflation ventilatory exchange (THRIVE) with conventional facemask ventilation technique for oxygenation in patients undergoing electroconvulsive therapy - a cross over study. Asian J Psychiatr. (2023) 88:103734. doi: 10.1016/j.ajp.2023.103734 37619421

[28] Deng CJ Nie S . Narrative review and consensus recommendations for the use of transnasal humidified rapid-insufflation ventilatory exchange in modified electroconvulsive therapy. Alpha Psychiatry. (2024) 25:282–9. doi: 10.5152/alphapsychiatry.2024.231463 PMC11117428 38798804

[29] Martinotti G Vita A Fagiolini A Maina G Bertolino A Dell’Osso B . Real-world experience of esketamine use to manage treatment-resistant depression: A multicentric study on safety and effectiveness (REAL-ESK study). J Affect Disord. (2022) 319:646–54. doi: 10.1016/j.jad.2022.09.043 36167246

[30] Rosso G d’Andrea G Barlati S Di Nicola M Andriola I Marcatili M . Esketamine treatment trajectory of patients with treatment-resistant depression in the mid and long-term run: data from REAL-ESK Study Group. Curr Neuropharmacol. (2025) 23:e1570159X337670. doi: 10.2174/011570159x337670241029062524 PMC12163464 39810448

[31] d’Andrea G Miuli A Pettorruso M Cavallotto C Marrangone C Cocco A . Exploring vortioxetine combination with intranasal esketamine: A feasible alternative to SSRI/SNRI? - Insights from the REAL-ESK study. J Affect Disord. (2024) 367:583–8. doi: 10.1016/j.jad.2024.09.004 39233241

[32] Alshammari TK . The ketamine antidepressant story: new insights. Molecules. (2020) 25. doi: 10.3390/molecules25235777 PMC7730956 33297563

[33] Berman RM Cappiello A Anand A Oren DA Heninger GR Charney DS . Antidepressant effects of ketamine in depressed patients. Biol Psychiatry. (2000) 47:351–4. doi: 10.1016/s0006-3223(99)00230-9 10686270

[34] Singh JB Fedgchin M Daly E Xi L Melman C De Bruecker G . Intravenous esketamine in adult treatment-resistant depression: a double-blind, double-randomization, placebo-controlled study. Biol Psychiatry. (2016) 80:424–31. doi: 10.1016/j.biopsych.2015.10.018 26707087

[35] Kryst J Kawalec P Mitoraj AM Pilc A Lasoń W Brzostek T . Efficacy of single and repeated administration of ketamine in unipolar and bipolar depression: a meta-analysis of randomized clinical trials. Pharmacol Rep. (2020) 72:543–62. doi: 10.1007/s43440-020-00097-z PMC7329804 32301056

[36] Zhan Y Zhang B Zhou Y Zheng W Liu W Wang C . A preliminary study of anti-suicidal efficacy of repeated ketamine infusions in depression with suicidal ideation. J Affect Disord. (2019) 251:205–12. doi: 10.1016/j.jad.2019.03.071 30927581

[37] Zhou Y Wang C Lan X Zheng W Li H Chao Z . The potential pro-cognitive effects with intravenous subanesthetic ketamine in adults with treatment-resistant major depressive or bipolar disorders and suicidality. J Psychiatr Res. (2021) 144:312–9. doi: 10.1016/j.jpsychires.2021.10.037 34715598

[38] Li W Zhou Y Liu W Wang C Lan X Zhang Z . Long-term outcomes of repeated ketamine infusions in patients with unipolar and bipolar depression: A naturalistic follow-up study. J Affect Disord. (2022) 300:172–8. doi: 10.1016/j.jad.2021.12.084 34952122

[39] Qiu L Chen X Fu J Chen X Wang X . Intravenous patient-controlled analgesia with esketamine improves early depressive symptoms in patients with postherpetic neuralgia: a single-center retrospective cohort study. BMC Psychiatry. (2024) 24:582. doi: 10.1186/s12888-024-06035-0 39192262 PMC11348644

[40] Andrade C . Ketamine for depression, 4: in what dose, at what rate, by what route, for how long, and at what frequency? J Clin Psychiatry. (2017) 78:e852–e7. doi: 10.4088/JCP.17f11738 28749092

[41] Kheirabadi D Kheirabadi GR Mirlohi Z Tarrahi MJ Norbaksh A . Comparison of rapid antidepressant and antisuicidal effects of intramuscular ketamine, oral ketamine, and electroconvulsive therapy in patients with major depressive disorder: a pilot study. J Clin Psychopharmacol. (2020) 40:588–93. doi: 10.1097/jcp.0000000000001289 33060432

[42] Nuñez NA Joseph B Pahwa M Seshadri A Prokop LJ Kung S . An update on the efficacy and tolerability of oral ketamine for major depression: a systematic review and meta-analysis. Psychopharmacol Bull. (2020) 50:137–63. 10.64719/pb.4376 PMC7511150 33012876

[43] Loo C Glozier N . Efficacy and safety of a 4-week course of repeated subcutaneous ketamine injections for treatment-resistant depression (KADS study): randomised double-blind active-controlled trial. Br J Psychiatry. (2023) 223:533–41. doi: 10.1192/bjp.2023.79 PMC10727911 38108319

[44] Wan-Jun Y Zhi-Long G Yuan-Yuan G Chao-Yuan C Zheng-Ze C Zi-Wei T . Comparative study of the efficacy and safety of remimazolam and midazolam for general anesthesia in elderly patients: a randomized controlled trial. Perioper Med (Lond). (2025) 14:53. doi: 10.1186/s13741-025-00525-9 40340759 PMC12060543

[45] Loo CK Gálvez V O’Keefe E Mitchell PB Hadzi-Pavlovic D Leyden J . Placebo-controlled pilot trial testing dose titration and intravenous, intramuscular and subcutaneous routes for ketamine in depression. Acta Psychiatr Scand. (2016) 134:48–56. doi: 10.1111/acps.12572 27028832

[46] Kryst J Kawalec P Pilc A . Efficacy and safety of intranasal esketamine for the treatment of major depressive disorder. Expert Opin Pharmacother. (2020) 21:9–20. doi: 10.1080/14656566.2019.1683161 31663783

[47] Yavi M Lee H . Ketamine treatment for depression: a review. Discov Ment Health. (2022) 2:9. doi: 10.1007/s44192-022-00012-3 35509843 PMC9010394

[48] Expert guidance group on clinical application of esketamine. guidance of clinical application experts of esketamine (In Chinese). Int J Anesth Resus. (2023) 44:785–93. doi: 10.3760/cma.j.cn321761-20230603-00852

[49] Guo X . Antidepressants can also be ‘ Ultra Rapid’. China Hosp CEO. (2024) 20:24–5.

[50] Chen M Yang X Liu C Li J Wang X . Comparative efficacy and cognitive function of magnetic seizure therapy vs. electroconvulsive therapy for major depressive disorder: a systematic review and meta-analysis. Transl Psychiatry. (2021) 11:437. doi: 10.1038/s41398-021-01560-y 34420033 PMC8380249

[51] Jiang J Zhang C Li C Chen Z Cao X Wang H . Magnetic seizure therapy for treatment-resistant depression. Cochrane Database Syst Rev. (2021) 6:Cd013528. doi: 10.1002/14651858.CD013528.pub2 34131914 PMC8205924

[52] Cai DB Yang XH Shi ZM Nie S Xu R Qin XD . Comparison of efficacy and safety of magnetic seizure therapy and electroconvulsive therapy for depression: a systematic review. J Pers Med. (2023) 13. doi: 10.3390/jpm13030449 PMC10057006 36983629

[53] Wang J Vila-Rodriguez F Ge R Gao S Gregory E Jiang W . Accelerated magnetic seizure therapy (aMST) for treatment of major depressive disorder: A pilot study. J Affect Disord. (2020) 264:215–20. doi: 10.1016/j.jad.2019.12.022 32056753

[54] Liu C Liu S Hu X Guo Z Xu Y . Fluctuations in resting motor threshold during electroconvulsive and magnetic seizure therapy. Int J Neurosci. (2024), 1–12. doi: 10.1080/00207454.2024.2401418 39230589

[55] Zhang J Ren Y . Shorter recovery times and better cognitive function-a comparative pilot study of magnetic seizure therapy and electroconvulsive therapy in patients with depressive episodes. Brain Behav. (2020) 10:e01900. doi: 10.1002/brb3.1900 33070479 PMC7749607

[56] Hoy KE Thomson RH Cherk M Yap KS Daskalakis ZJ Fitzgerald PB . Effect of magnetic seizure therapy on regional brain glucose metabolism in major depression. Psychiatry Res. (2013) 211:169–75. doi: 10.1016/j.pscychresns.2012.08.003 23149039

[57] Kayser S Bewernick BH Matusch A Hurlemann R Soehle M Schlaepfer TE . Magnetic seizure therapy in treatment-resistant depression: clinical, neuropsychological and metabolic effects. Psychol Med. (2015) 45:1073–92. doi: 10.1017/s0033291714002244 25420474

[58] Ge R Gregory E Wang J Ainsworth N Jian W Yang C . Magnetic seizure therapy is associated with functional and structural brain changes in MDD: therapeutic versus side effect correlates. J Affect Disord. (2021) 286:40–8. doi: 10.1016/j.jad.2021.02.051 33676262

[59] Atluri S Wong W Moreno S Blumberger DM Daskalakis ZJ Farzan F . Selective modulation of brain network dynamics by seizure therapy in treatment-resistant depression. NeuroImage Clin. (2018) 20:1176–90. doi: 10.1016/j.nicl.2018.10.015 PMC6214861 30388600

[60] Farzan F Atluri S Mei Y Moreno S Levinson AJ Blumberger DM . Brain temporal complexity in explaining the therapeutic and cognitive effects of seizure therapy. Brain. (2017) 140:1011–25. doi: 10.1093/brain/awx030 28335039

[61] Hill AT Zomorrodi R Hadas I Farzan F Voineskos D Throop A . Resting-state electroencephalographic functional network alterations in major depressive disorder following magnetic seizure therapy. Prog Neuropsychopharmacol Biol Psychiatry. (2021) 108:110082. doi: 10.1016/j.pnpbp.2020.110082 32853716

[62] Smith SE Kosik EL van Engen Q Kohn J Hill AT . Magnetic seizure therapy and electroconvulsive therapy increase aperiodic activity. Transl Psychiatry. (2023) 13:347. doi: 10.1038/s41398-023-02631-y 37968260 PMC10651875

[63] Sun Y Blumberger DM Mulsant BH Rajji TK Fitzgerald PB Barr MS . Magnetic seizure therapy reduces suicidal ideation and produces neuroplasticity in treatment-resistant depression. Transl Psychiatry. (2018) 8:253. doi: 10.1038/s41398-018-0302-8 30470735 PMC6251931

[64] Johnson KA Baig M Ramsey D Lisanby SH Avery D McDonald WM . Prefrontal rTMS for treating depression: location and intensity results from the OPT-TMS multi-site clinical trial. Brain Stimul. (2013) 6:108–17. doi: 10.1016/j.brs.2012.02.003 PMC3573221 22465743

[65] Berlim MT van den Eynde F Tovar-Perdomo S Daskalakis ZJ . Response, remission and drop-out rates following high-frequency repetitive transcranial magnetic stimulation (rTMS) for treating major depression: a systematic review and meta-analysis of randomized, double-blind and sham-controlled trials. Psychol Med. (2014) 44:225–39. doi: 10.1017/s0033291713000512 23507264

[66] Lan XJ Yang XH Qin ZJ Cai DB Liu QM Mai JX . Efficacy and safety of intermittent theta burst stimulation versus high-frequency repetitive transcranial magnetic stimulation for patients with treatment-resistant depression: a systematic review. Front Psychiatry. (2023) 14:1244289. doi: 10.3389/fpsyt.2023.1244289 37583841 PMC10423820

[67] Blumberger DM Vila-Rodriguez F Thorpe KE Feffer K Noda Y Giacobbe P . Effectiveness of theta burst versus high-frequency repetitive transcranial magnetic stimulation in patients with depression (THREE-D): a randomised non-inferiority trial. Lancet. (2018) 391:1683–92. doi: 10.1016/s0140-6736(18)30295-2 29726344

[68] Cheng CM Li CT Tsai SJ . Current updates on newer forms of transcranial magnetic stimulation in major depression. Adv Exp Med Biol. (2021) 1305:333–49. doi: 10.1007/978-981-33-6044-0_18 33834408

[69] Huang D Zhong S Song X Zhang R Lai S Jia Y . Effect of novel accelerated intermittent theta burst stimulation on suicidal ideation in adolescent patients with major depressive episode: a randomised clinical trial. Gen Psychiatr. (2024) 37:e101394. doi: 10.1136/gpsych-2023-101394 38665940 PMC11043680

[70] Neuteboom D Zantvoord JB Goya-Maldonado R Wilkening J Dols A van Exel E . Accelerated intermittent theta burst stimulation in major depressive disorder: A systematic review. Psychiatry Res. (2023) 327:115429. doi: 10.1016/j.psychres.2023.115429 37625365

[71] Lan XJ Cai DB Liu QM Qin ZJ Pridmore S Zheng W . Stanford neuromodulation therapy for treatment-resistant depression: a systematic review. Front Psychiatry. (2023) 14:1290364. doi: 10.3389/fpsyt.2023.1290364 38161728 PMC10756664

[72] Cole EJ Phillips AL Bentzley BS . Stanford neuromodulation therapy (SNT): a double-blind randomized controlled trial. Am J Psychiatry. (2022) 179:132–41. doi: 10.1176/appi.ajp.2021.20101429 34711062

[73] Cole EJ Stimpson KH Bentzley BS Gulser M Cherian K Tischler C . Stanford accelerated intelligent neuromodulation therapy for treatment-resistant depression. Am J Psychiatry. (2020) 177:716–26. doi: 10.1176/appi.ajp.2019.19070720 32252538

[74] Williams NR Sudheimer KD Bentzley BS Pannu J Stimpson KH Duvio D . High-dose spaced theta-burst TMS as a rapid-acting antidepressant in highly refractory depression. Brain. (2018) 141:e18. doi: 10.1093/brain/awx379 29415152 PMC5837258

[75] Li B Zhao N Tang N Friston KJ . Targeting suicidal ideation in major depressive disorder with MRI-navigated Stanford accelerated intelligent neuromodulation therapy. Transl Psychiatry. (2024) 14:21. doi: 10.1038/s41398-023-02707-9 38199983 PMC10781692

[76] Tang N Chen Y Wang Y Sun C Liu C Wu D . A preliminary study of precise treatment for major depression patients with suicide ideation by individualized targeted robot assisted Stanford accelerated intelligent neuromodulation therapy (In Chinese). Chin J Psychiatry. (2022) 55:14–23. doi: 10.3760/cma.j.cn113661-20210527-00173

[77] Zhang Y Mu Y Li X Sun C Ma X Li S . Improved interhemispheric functional connectivity in postpartum depression disorder: associations with individual target-transcranial magnetic stimulation treatment effects. Front Psychiatry. (2022) 13:859453. doi: 10.3389/fpsyt.2022.859453 35370853 PMC8964485

[78] Liu X Li P Xia Y Yuan S Chen C Xie K . Efficacy study of intermittent theta burst stimulation(iTBS) in the treatment of adolescents with affective disorders. J Affect Disord. (2025) 373:284–90. doi: 10.1016/j.jad.2025.01.002 39761754

[79] Zheng W Zhou YL Liu WJ Wang CY Zhan YN Li HQ . Neurocognitive performance and repeated-dose intravenous ketamine in major depressive disorder. J Affect Disord. (2019) 246:241–7. doi: 10.1016/j.jad.2018.12.005 30590286

[80] Zhang W Ma N . China’s national comprehensive management pilot project for mental health. BJPsych Int. (2017) 14:44–6. doi: 10.1192/s2056474000001781 PMC5618815 29093939

[81] Short B Dong V Gálvez V Vulovic V Martin D Bayes AJ . Development of the ketamine side effect tool (KSET). J Affect Disord. (2020) 266:615–20. doi: 10.1016/j.jad.2020.01.120 PMC7693479 32056935

[82] Petrucci ABC Fernandes JVA Reis IA da Silva GHS Recla BMF de Mendonça JC . Ketamine versus electroconvulsive therapy for major depressive episode: An updated systematic review and non-inferiority meta-analysis. Psychiatry Res. (2024) 339:115994. doi: 10.1016/j.psychres.2024.115994 38865906

[83] de ASMD Gauer LE Teixeira G Fonseca da Silva AC Cavalcanti S Quevedo J . Efficacy and adverse effects of ketamine versus electroconvulsive therapy for major depressive disorder: A systematic review and meta-analysis. J Affect Disord. (2023) 330:227–38. doi: 10.1016/j.jad.2023.02.152 PMC10497186 36907464

[84] Abuhelwa AY Somogyi AA . Population pharmacokinetics and pharmacodynamics of the therapeutic and adverse effects of ketamine in patients with treatment-refractory depression. Clin Pharmacol Ther. (2022) 112:720–9. doi: 10.1002/cpt.2640 PMC9540482 35560226

[85] Peltoniemi MA Hagelberg NM Olkkola KT Saari TI . Ketamine: a review of clinical pharmacokinetics and pharmacodynamics in anesthesia and pain therapy. Clin Pharmacokinet. (2016) 55:1059–77. doi: 10.1007/s40262-016-0383-6 27028535

[86] Deng ZD Luber B McClintock SM Weiner RD Husain MM Lisanby SH . Clinical outcomes of magnetic seizure therapy vs electroconvulsive therapy for major depressive episode: a randomized clinical trial. JAMA Psychiatry. (2024) 81:240–9. doi: 10.1001/jamapsychiatry.2023.4599 PMC10701670 38055283

[87] Pettorruso M Guidotti R d’Andrea G De Risio L D’Andrea A Chiappini S . Predicting outcome with intranasal esketamine treatment: a machine-learning, three-month study in treatment-resistant depression (ESK-LEARNING). Psychiatry Res. (2023) 327:115378. doi: 10.1016/j.psychres.2023.115378 37574600

[88] Ma N Chen R Zhang W Wang Y Bai Y Su R . The mental health resources in Chinese mainland by 2020. Chin J Psychiatry. (2022) 55:459–68. doi: 10.3760/cma.j.cn113661-20220617-00158

[89] Lu J Xu X Huang Y Li T Ma C Xu G . Prevalence of depressive disorders and treatment in China: a cross-sectional epidemiological study. Lancet Psychiatry. (2021) 8:981–90. doi: 10.1016/s2215-0366(21)00251-0 34559991

