# Correction: Rapid treatment center for depression in China: constructive reflections and transnational implications

**DOI:** 10.3389/fpsyt.2025.1725742

**Published:** 2025-11-07

**Authors:** Zhan-Ming Shi, Xing-Bing Huang, Yan-Ling Zhou, Yu-Ping Ning, Gabor S. Ungvari, Yu-Tao Xiang, Wei Zheng

**Affiliations:** 1 Department of Psychiatry, Chongqing Jiangbei Second Hospital, Chongqing Jiangbei Mental Health Center, Chongqing, China; 2 Department of Psychiatry, The Affiliated Brain Hospital, Guangzhou Medical University, Guangzhou, China; 3 Key Laboratory of Neurogenetics and Channelopathies of Guangdong Province and the Ministry of Education of China, Guangzhou Medical University, Guangzhou, China; 4 Section of Psychiatry, University of Notre Dame Australia, Fremantle, WA, Australia; 5 Division of Psychiatry, School of Medicine, University of Western Australia, Perth, WA, Australia; 6 Unit of Psychiatry, Department of Public Health and Medicinal Administration, & Institute of Translational Medicine, Faculty of Health Sciences, University of Macau, Macao, Macao SAR, China; 7 Centre for Cognitive and Brain Sciences, University of Macau, Macao, Macao SAR, China

**Keywords:** major depressive disorder, electroconvulsive therapy, ketamine, esketamine, esketamine nasal spray, magnetic seizure therapy, Stanford Neuromodulation Therapy, rapid treatment center

A correction has been made to the section **3 Actionable recommendations**, *3.1 Electroconvulsive therapy*, Paragraph 1. The sentence previously stated:

“Because of its relatively moderate efficacy rate of approximately 60.0% (10) and known neurocognitive side effects (11), there are ongoing efforts aiming at refining the clinical application of ECT from various perspectives, including anesthetic selection, stimulation dosage, electrode placement and apnoeic oxygenation.”

The corrected sentence appears below:

“Because of its relatively moderate efficacy rate of 58% and 70%, for those with and without medication failure, respectively (10) and known neurocognitive side effects (11), there are ongoing efforts aiming at refining the clinical application of ECT from various perspectives, including anesthetic selection, stimulation dosage, electrode placement and apnoeic oxygenation.”

A correction has been made to the section **3 Actionable recommendations**, *3.3 Magnetic seizure therapy*, Paragraph 1. The sentence previously stated:

“Capitalizing on its advantageous profile of reduced neurocognitive side effects and comparable antidepressant efficacy to ECT (52), a team at Beijing Anding Hospital of Capital Medical University, have adopted an accelerated MST protocol termed daily MST, demonstrating rapid antidepressant effects (53).”

The corrected sentence appears below:

“Capitalizing on its advantageous profile of comparable antidepressant efficacy to ECT (52), a team at Beijing Anding Hospital of Capital Medical University have adopted an accelerated MST protocol termed daily MST, demonstrating rapid antidepressant effects (53).”

A correction has been made to the section **4 Discussion**, *4.2 Comparisons of rapid antidepressant treatments*, Paragraph 1. The sentences previously stated:

“A recent meta-analysis of six RCTs comparing the efficacy and safety of ECT with ketamine revealed no significant disparity between the two treatments (82). Conversely, another meta-analysis (83) concluded that ketamine, regardless of its administration route, surpassed ECT in effectiveness for MDD patients.”

The corrected sentences appear below:

“A recent meta-analysis supported the use of ECT over ketamine for inpatients (82). Conversely, another meta-analysis of RCTs comparing the efficacy of ECT with ketamine revealed no significant disparity between the two treatments (83).”

A correction has been made to the section **4 Discussion**, *4.3 General/systemic deficiencies*, Paragraph 2. The sentence previously stated:

“In rural regions, only 5.1% of patients with MDD received any healthcare treatment, compared to 8.9% in urban areas (89).”

The corrected sentence appears below:

“In rural regions, only 5.1% of patients with depressive disorders received any form of healthcare treatment, compared to 8.9% in urban areas (89).”

The original version of this article has been updated.

